# "Comprehensive Care" Concept in Nursing: Systematic Review*

**DOI:** 10.17533/udea.iee.v40n3e05

**Published:** 2023-02-09

**Authors:** Alina Renghea, Miguel Angel Cuevas-Budhart, Hugo Yébenes-Revuelto, Mercedes Gómez del Pulgar, María Teresa Iglesias-López

**Affiliations:** 1 Enfermera, Candidata a Doctora. Profesora. Facultad de Ciencias de la Salud. Universidad Francisco de Vitoria, España , Email: a.renguea@ufv.es Universidad Francisco de Vitoria Facultad de Ciencias de la Salud Universidad Francisco de Vitoria Spain a.renguea@ufv.es; 2 .Enfermero, Candidato a Doctor. Unidad de Investigación Médica en Enfermedades Nefrológicas, IMSS, México. angel_budhart@hotmail.com Unidad de Investigación Médica en Enfermedades Nefrológicas IMSS México angel_budhart@hotmail.com; 3 .Biólogo, Doctor. Profesor. Facultad de Ciencias de la Salud. Universidad Francisco de Vitoria, España , Email: hugo.yebenes@ufv.es Universidad Francisco de Vitoria Facultad de Ciencias de la Salud Universidad Francisco de Vitoria Spain hugo.yebenes@ufv.es; 4 .Enfermera, Doctora. Profesora. Facultad de Ciencias de la Salud. Universidad Francisco de Vitoria España , Email: m.gomezdelpulgar@ufv.es Universidad Francisco de Vitoria Facultad de Ciencias de la Salud Universidad Francisco de Vitoria Spain m.gomezdelpulgar@ufv.es; 5.Farmaceútica, Doctora. Profesora. Facultad de Ciencias de la Salud. Universidad Francisco de Vitoria España , Email: m.iglesias.prof@ufv.es. Corresponding author Universidad Francisco de Vitoria Facultad de Ciencias de la Salud Universidad Francisco de Vitoria Spain m.iglesias.prof@ufv.es

**Keywords:** comprehensive health care, nursing care, systematic review., atención integral de salud, atención de enfermería, revisión sistemática., assistência integral à saúde, cuidados de enfermagem, revisão sistemática.

## Abstract

**Introduction.:**

Integrated health care is a concept widely used in the planning and organisation of nursing care. It is a highly topical concept, but at the same time it is deeply rooted in the theory and models of Nursing right from its inception as a science. There is no clear, agreed definition that describes it.

**Objective.:**

To systematise the knowledge available on the concept of "comprehensive care" in Nursing from the point of view of nursing care, its domains and characteristics.

**Methods.:**

A literature search has been carried out in several languages (Spanish, Portuguese, English and Romanian) in the databases Web of Science, Scopus, Medline, PubMed, Cochrane and Dialnet, covering the period between 2013 and 2019. The search terms used were: *comprehensive health care, health and nursing.* Prospero register 170327.

**Resultados.:**

Sixteen documents were identified, which grouped 8 countries, mainly Brazil, being the country with the highest output on this context- 10 documents were found within the qualitative paradigm and 6 quantitative ones. The concept "Comprehensive Care" is commonly used to refer to comprehensive nursing care techniques, protocols, programmes and plans, covering care in all aspects of the individual as a complement to or independent of the clinical needs arising from health care.

**Conclusion.:**

The definition of features pertaining to the concept "Comprehensive Care" encourages the use and standardisation of nursing care plans, improving patient follow-up, the detection of new risk factors, complications and new health problems not related to the reason for admission. This increases the capacity for prevention and improves the patient’s quality of life, and their primary and/or family caregivers, which translates into lower costs in the health system.

## Resumen

**Antecedentes.:**

El cuidado integral en términos de salud es un concepto muy utilizado en la planificación y organización de los cuidados enfermeros. Es un concepto muy actual, pero a la vez muy arraigado en la teoría y los modelos de Enfermería desde sus principios cómo ciencia. No obstante, no existe una definición clara, consensuada que lo describa.

**Objetivo.:**

Sistematizar los conocimientos disponibles sobre el concepto de "cuidado integral" en Enfermería desde el punto de vista del cuidado de enfermería, sus dominios y características.

**Métodos.:**

Revisión sistemática de la información en idioma inglés, español, romaní y portugués en las siguientes bases de datos: Web of Science, Scopus, Medline, PubMed, Cochrane y Dialnet. Dicha búsqueda abarcó el período comprendido entre 2013 y 2020. Los términos usados fueron: *comprehensive health care, health and nursing*. Prospero register 170327.

**Resultados.:**

Se identificaron dieciséis documentos, que agruparon a 8 países, principalmente Brasil, siendo el país con mayor producción en este contexto, se encontraron 10 documentos dentro del paradigma cualitativo y 4 cuantitativos. El concepto "Cuidados Integrales" es comúnmente utilizado para referirse a las técnicas, protocolos, programas y planes de cuidados integrales de enfermería, que abarcan el cuidado en todos los aspectos del individuo como complemento o independiente de las necesidades clínicas derivadas del cuidado de la salud.

**Conclusión.:**

La definición de características propias del concepto "Cuidados Integrales" favorece la utilización y estandarización de los planes de cuidados de enfermería, mejorando el seguimiento de los pacientes, detectando nuevos factores de riesgo, complicaciones y nuevos problemas de salud no relacionados con el motivo de ingreso. Esto aumenta la capacidad de prevención y mejora la calidad de vida del paciente y de sus cuidadores principales y familiares, lo que se traduce en menores costes en el sistema sanitario.

## Resumo

**Antecedentes.:**

O cuidado integral em saúde é um conceito amplamente utilizado no planejamento e organização da assistência de enfermagem. É um conceito muito atual, mas ao mesmo tempo profundamente enraizado na teoria e nos modelos de Enfermagem desde seus primórdios como ciência. No entanto, não existe uma definição clara e consensual que a descreva.

**Objetivo.:**

Sistematizar o conhecimento disponível sobre o conceito de "cuidado integral" em Enfermagem sob o ponto de vista do cuidado de enfermagem, seus domínios e características.

**Métodos.:**

Revisão sistemática de informações em inglês, espanhol, romani e português nas seguintes bases de dados: Web of Science, Scopus, Medline, PubMed, Cochrane e Dialnet. Essa busca abrangeu o período entre 2013 e 2020. Os termos utilizados foram: atenção integral à saúde, saúde. e enfermagem. Registro Prospero 170327

**Resultados.:**

Foram identificados 16 documentos, que agruparam 8 países, principalmente o Brasil, sendo o país com maior produção neste contexto, foram encontrados 10 documentos dentro do paradigma qualitativo e 4 quantitativos. O conceito “Cuidado Integral” é comumente utilizado para se referir às técnicas, protocolos, programas e planos de assistência integral de enfermagem, que englobam o cuidado em todos os aspectos do indivíduo como complemento ou independente das necessidades clínicas derivadas do cuidado em saúde.

**Conclusão.:**

A definição das características do conceito "Cuidado Integral" favorece a utilização e padronização dos planos de assistência de enfermagem, melhorando o acompanhamento do paciente, detectando novos fatores de risco, complicações e novos problemas de saúde não relacionados ao motivo da internação. Isso aumenta a capacidade de prevenção e melhora a qualidade de vida do paciente e de seus cuidadores primários e familiares, o que se traduz em menores custos no sistema de saúde.

## Introduction

In recent years, providing quality care has been a priority issue for the health system, managers and health professionals.(1) Therefore, comprehensive care is the concept most widely used, with the aim of describing health care as a complete service, technically correct, humanised and individual-friendly.(2) However, the concept lacks defining criteria in healthcare in general and in nursing in particular, as our profession is very broad and has competencies in all healthcare areas. Caring has been strictly linked to nursing since the beginning of the profession and has been perfected and professionalised as nursing models have evolved. These are the basis of the general guidelines for clinical practice together with the nursing work method (the Nursing Care Process and its implementing tool- The Care Plan). In the research field, nursing models allow knowledge to be organised and direct researchers towards those health problems that need to be known in greater depth.(3,4)

According to the American Nursing Association, the nursing process is considered as a standard for nursing practice; its importance has demanded substantial changes in its stages, favouring the development of the profession as a scientific discipline, which in turn has increased the quality of care.(5,6) The nursing process consists of five stages, which are closely related- assessment, diagnosis, planning, implementation and evaluation. For this study, we will refer only to the first two stages. The nursing process is designed to respond to the needs of the people we care for and to restore the patient's health and autonomy. It has become the nurses' most valuable tool. Today, the Nursing Care Plan provides the necessary support for the implementation of nursing care. On this basis, nursing care must be applied to all persons needing it and in all its aspects, both in the family and in the patient’s social and environmental settings. Therefore, the nursing model is based on care, both in theory and in practice, which has been enriched and completed over time. It is noteworthy that by identifying the concept of comprehensive care “foundations are laid to achieve optimal results in care, favouring comprehensive care for the individual in all areas- biophysiological, psychological, social and spiritual.(7-9)

Under this premise, this study answers the following research question: What is the best scientific evidence available on the concept of "comprehensive health care" in Nursing care from the point of view of nursing care, its domains, and its characteristics? The definition of the concept starts from an analogy between concepts with the purpose of clarifying logical points and establishing relevant concepts of the domain that unite them, for which the exhaustive analysis of the information is used as a research technique, with the intention that this definition serves as a new line of research and that has a greater level of depth. In order to have arguments to support this assessment, the specific objective of this review is to systematize the available knowledge on the "comprehensive health care" concept in nursing from standpoint of the point of view of nursing care, its domains, and its characteristics.

## Methods

A systematic review was conducted as a scientific method for the identification, collection, evaluation and synthesis of existing scientific evidence.(9) However, an integrative review method was also used, as it provides a systematic and rigorous process designed to enable a comprehensive understanding of the context.(10) The search was standardised and systematised to obtain the most relevant information about the concept of "Comprehensive Care". It is worthy to mention the significance of defining the concept in the nursing care framework through the care plan, to measure the quality of service in nursing, and to detect improvement areas. 

*Eligibility Criteria.* All works were considered eligible, both qualitative and quantitative, of type retrospective, prospective, and cross-sectional studies, in addition, these studies should have been conducted by Nursing and with free access. To ensure that documents were only reviewed in the context of Nursing, exclusion criteria were required to further refine the search and the publications found during the database search. Thus, all studies that do not contain the concept of comprehensive health care in the title or the abstract, as well as studies that do not refer to comprehensive health care oriented to nursing practice, were excluded. 

*Search Strategy.* To search for information, only observational scientific articles were included, whether transversal or longitudinal, published from January 2013 to 2020, available in open access, published in English, Spanish, Romanian and Portuguese. 

The databases consulted were Web of Science, Scopus, Medline, PubMed, Cochrane, and Dialnet. The keywords used were comprehensive health care, health. and nursing, following the criteria established in Prisma.(11) The Boolean operators used were the intersection: AND to establish the logical operations between concepts, OR to retrieve documents containing at least one of the specified arguments, and NOT to indicate that the keyword before the operator must appear, but not the one after it: Comprehensive health care AND health care AND Nursing NOT holistic care. 

*Screening Process.* All publications included were reviewed in full by the authors. Data extraction included country identification, study design, and key findings. Moreover, a review of publication type was conducted. If it was a research publication, then the type of study, sample, and purpose of the publication was recorded. For the selection process, duplicates were removed, followed by a check of the titles and abstracts by selecting documents that could be relevant to the study successively in the first screening. The presence of the ''comprehensive care'' concept and a subsequent reference to Nursing were sought in the title and abstract of each article. The evaluation of the methodological quality of the articles included was carried out through a tool for the evaluation of studies resulting from qualitative research.(12) For the quantitative studies, the STROBE (14,15) report on observational studies in epidemiology was used. 

This review has been registered in PROSPERO with reference number 170327. 

**Figure 1:**
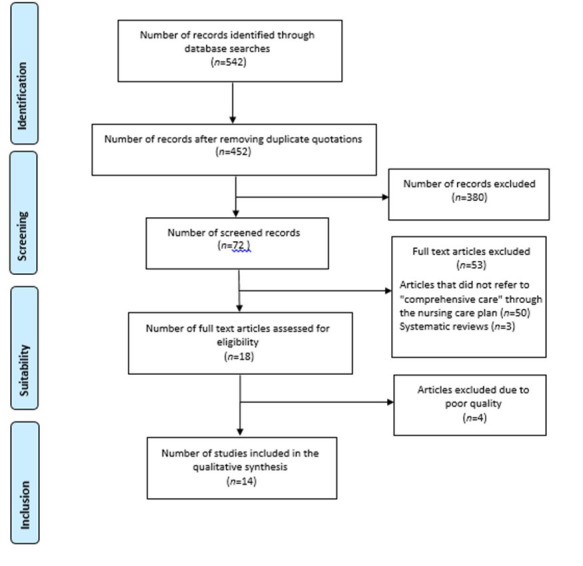
Flow-chart of Prisma diagram

## Results

After removing duplicates, the primary search reviewed 452 scientific articles from databases such as Web of Science, Scopus, Medline, PubMed, Cochrane and Dialnet. These were selected according to the title, abstract and full text, thereby limiting in each stage. The main reasons for rejection were that the studies did not provide a description of comprehensive care within the title and abstract, and that the concept was not found within clinical practice. The final analysis of the studies included in the qualitative synthesis showed 14 articles (Figure 1). 

In accordance with the inclusion criteria established in the first screening of this search in the aforementioned databases, 72 references were found and 380 were excluded due to the type of methodological design, and because they did not meet the inclusion criteria related to the review’s objective, therefore, as a result of this process, 72 results meeting the defined criteria were selected. It was decided to carry out a second screening in order to select those articles that were available in full text and included the concept of comprehensive care specifically through nursing care plans or nursing interventions, and which were not literature reviews.

After this second screening, 18 potentially relevant references were selected to assess the methodological design in terms of the inclusion criteria established in the second screening. Eight different countries were identified from the 14 documents to be jointly evaluated (Table 1). The country with the highest scientific output in this context is Brazil with 8 documents (57%),(7,9,16-22) USA,(23) Australia,(24) Iran,(25) Ghana,(8) Colombia,(26) and India(27) with one study each per country. 

**Table 1:** Summary of selected articles

Reference 7. Rocha RCNP, Pereira ER, Silva RMCRA, Medeiros AYBBV, Refrande SM, Refrande NA. Spiritual needs experienced by the patient’s family caregiver under Oncology palliative care. Rev. Bras. Enferm. 2018; 71:2635-42
Country: Brazil
Study design: Qualitative
Objective: To understand the spiritual needs of the patients' family caregiver under Oncology palliative care.
Sample: 20
Type of population: Family members, primary caregivers.
Key results: It concludes that spiritual care is part of comprehensive care, and it is important in the life of the patient; family members underestimate it, and nurses are expected to include it in the patient care.
Evaluation of methodological quality: Moderate = 9.
Reference 8. Agyeman-Yeboah J, Korsah K, Okrah J. Factors that influence the clinical utilization of the nursing process at a hospital in Accra, Ghana. BMC Nursing. 2017;16(1):30.
Country: Ghana.
Study design: Qualitative
Objective: The purpose of this research study was to explore the various factors that influence the utilization of this nursing process.
Sample: 10
Type of population: Nurses, supervising nurses and specialist nurses at the 37^th^ Military Hospital, Ghana
Key results: The result shows that the care plan meets the requirements of comprehensive care in all the individual’s areas, that it is not very operational and known within the nursing community, which is why it is not used as often as it should be.
Evaluation of methodological quality: Moderate = 9.
Reference 9. Galvão TLA, Oliveira KKD, Maia CAAS, Miranda FAN. Assistência à pessoa com Parkinson no âmbito da estratégia de saúde da família. Rev. Pesqui (Univ. Estado Rio J., Online). 2016; 8(4):5101-7.
Country: Brazil
Study design: Qualitative
Objective: To analyse the conceptions that the bearer of Parkinson's disease (PD) holds about the comprehensive care provided by the nurse.
Sample: 5
Type of population: Patients
Key results: It concludes that nurses do not support the personalised care plan for the patient, and do not include the family in the caring process, but do take responsibility for prevention, promotion and health education.
Evaluation of methodological quality: Moderate = 9.
Reference 16. Nóia T de C, Evangelista Sant’Ana RS, dos Santos ADS, Oliveira S de C, Veras SMCB, Lopes-Júnior LC. Coping with the diagnosis and hospitalization of a child with childhood cancer. Invest. Educ. Enferm. 2015; 33(3):465-72.
Country: Brazil
Study design: Qualitative
Objective: Find out how family members cope with hospitalization due to the diagnosis of childhood cancer.
Sample: 10
Type of population: Family caregivers of children with cancer
Key results: Caring for children with cancer disrupts family life in all its aspects. Comprehensive care is needed in all areas and needs of the patient. It concludes that a nurse must provide humanised and sensitive comprehensive care.
Evaluation of methodological quality: Moderate = 11.
Reference 17. Azevedo A, Scarparo A, Chaves L. Nurses’ care and management actions in emergency trauma cases. Invest. Educ. Enferm. 2013; 31(1):36-43.
Country: Brazil
Study design: Qualitative
Objective: To analyse the nurses care and management actions in an emergency trauma hospital unit.
Sample: 11
Type of population: Nurses
Key results: It concludes that the nursing practice in the trauma emergency section comes close to the comprehensive care approach by complementing the patient care management with the services and resources management.
Evaluation of methodological quality: Moderate = 8.
Reference 18. Cattani AN, Foggiato de Siqueira D, Gomes Terra M. The care towards individuals in a Psychosocial Intervention Unit: meanings assigned by the nursing team. Rev. Pesqui (Univ. Estado Rio J., Online): 2018; 10(4):951-7.
Country: Brazil
Study design: Qualitative
Objective: To understand the meanings attributed by the nursing team to the care provided to people hospitalized in a psychosocial hospitalization unit of a public teaching hospital in Rio Grande do Sul, Brazil.
Sample: 15
Type of population: Nurses
Key results: It concludes that comprehensive care must be provided for all the individual’s needs, and that this care extends beyond discharge and includes the family, and that nursing is a group whose objective as a profession is the comprehensive care of the individual.
Evaluation of methodological quality: Optimal = 12.
Reference 20. de Carvalho Furtado M, Falleiros de Mello D, Coelho Pina J, Batistela Vicente J, Remundini de Lima P, Dias Rezende V. Nurses’ actions and articulations in child care in primary health care. Texto Contexto Enferm. 2017; 27(1):e0930016.
Country: Brazil
Study design: Qualitative
Objective: Understand how nursing care for children under five is configured in Family Health Units, focusing on comprehensive care.
Sample: 26
Type of population: Review of paediatric NCPs and during nursing interventions in medical consultation
Key results: It recommends meeting the requirement to practice comprehensive care, including the family, and also children. This recommendation, moreover, coincides with current public health policies.
Evaluation of methodological quality: Optimal = 12.
Reference 22. Rocha MGL, Linard AG. View of Women perceptions on the comprehensive care in the context of prevention of cervical cancer. Rev. Rene. 2016; 17(5):676-83.
Country: Brazil
Study design: Qualitative
Objective: To know the perceptions of women on the comprehensive health care in the context of prevention of cervical cancer.
Sample: 34
Type of population: It concludes that comprehensive care improves the quality of life of patients, avoids complications and detects potential health problems more accurately, but that implementation depends on the nursing professionals.
Evaluation of methodological quality: Moderate = 11.
Reference 23. Britton M, Ouellet K, Gawel E, Hodshon S. Care Transitions Between Hospitals and Skilled Nursing Facilities: Perspectives of Sending and Receiving Providers. Jt. Comm. J. Qual. Patient Saf. 2017; 43(11):565-72.
Country: EEUU
Study design: Qualitative
Objective: To identify the perspectives of sending and receiving providers regarding care transitions between the hospital and the SNF
Sample: 31
Type of population: Nurses, supervisors, doctors, social workers and patients
Key results: It concludes that, due to the complexity of chronic patients, communication with the patient and his or her psychosocial needs must be improved.
Evaluation of methodological quality: Optimal = 22.
Reference 24. Peel NM, Hornby-Turner YC, Henderson A, Hubbard RE, Gray LC. Prevalence and Impact of Functional and Psychosocial Problems in Hospitalized Adults: A Prospective Cohort Study. J. Am. Med. Dir. Assoc. 2019; 20(10):1294-1299.e1.
Country: Australia
Study design: Cross-sectional, descriptive
Objective: To investigate the prevalence of functional and psychosocial problems in hospitalized adults, to compare prevalence rates across age groups, and to assess their impact on discharge outcomes
Sample: 910
Type of population: Patients
Key results: It concludes that due to the high prevalence of psychosocial problems in the studied population, it is recommended that this type of care be included in nursing care plans, as part of comprehensive inpatient and post-discharge care.
Evaluation of methodological quality: Optimal = 20
Reference 25. Kavosi A, Taghiabadi M, Mohammadi G, Yazdi K, Shirdelzadeh S, Nasiri H, et al. Nursing manager’s attitude toward spirituality and spiritual care in Khorasan Razavi Province hospitals in 2016. Electron. Physician. 2018; 10(3):6571.
Country: Irán
Study design: Cross-sectional, analytical
Objective: The aim of this study was to determine nursing managers' attitude to spirituality and spiritual care in hospitals in Khorasan Razavi Province in 2016
Sample: 110
Type of population: Hospital management nurses in Khorasan Razavi province, Iran.
Key results: The result showed significant differences between the attitude towards spirituality and spiritual care and the gender experience, age and work in nursing management (*p* <0.05).
Evaluation of methodological quality: Optimal = 20.
Reference 26. Guerrero NS, Tobos LS. Care of an ostomized child: changes in family. Av. Enferm. 2013; 31(1):59-71.
Country: Colombia
Study design: Quantitative, Descriptive
Objective: Describe the impact of having an ostomised child on family dynamics, lifestyle and care preservation and traditional norms in the population of the Comprehensive Care Program for Ostomised Children and Adolescents and their Families at the University School of Nursing Nacional de Colombia at the Hospital de la Misericordia Foundation, Bogotá, Colombia.
Sample: 94
Type of population: Parents of children with an ostomy
Key results: It concludes that the care of a child with ostomy impacts all aspects of daily life. It emphasises the importance of caring for negative emotions and religion in the child's life. Furthermore, such care is part of a comprehensive health care strategy and it improves the child's quality of life.
Evaluation of methodological quality: Optimal = 21.
Reference 27. Pai R, Ongole R, Banerjee S. Oral care in cancer nursing: Practice and barriers. Indian J. Dent. Res. 2019; 30(2):226-30.
Country: Brazil
Study design: Cross-sectional, descriptive
Objective: To determine the nurses' practice and barriers regarding oral care in cancer patients undergoing chemotherapy and radiation therapy.
Sample: 159
Type of population: Nurses
Key results: It reminds nurses that oral cavity care is part of comprehensive care and should be included in the patient's regular care plan.
Evaluation of methodological quality: Optimal = 21.
Reference 32. Martínez LCV, Vidal LIE, Figueras MP, Hurtado JCT. Evaluating and promoting competencies for social entrepreneurship in university subjects. Rev. Estud. Cooperativos. 2019; 131:199-223.
Country: Brazil
Study design: Qualitative
Objective: Describe care needs and demands that mark the discursive practices of ostomised clients and family members and discuss guidelines for a comprehensive care program to ostomised clients and their families, organized by macrosociological categories.
Sample: 17
Type of population: Ostomised patients. Family members
Key results: Due to the complexity of the ostomised patient, it is important to make a special effort to address his or her psychosocial and spiritual needs, in order to improve his or her quality of life through comprehensive nursing care.
Evaluation of methodological quality: Optimal = 11.

Concerning the methodology used in the studies, the qualitative paradigm prevailed with(7,8,15-17,21,24-26,29) 62%, followed by the quantitative, three corresponding to the descriptive cross-sectional method(18,21-22) and a single observational, analytical cross-sectional article.(19) 

The average population used was 103 patients, with a minimum of 5 and a maximum of 910. The population targeted by these studies was made up of various health professionals and specifically Nurses, Patients, Caregivers and family members. The concept of comprehensive care is referred to in most articles as complete care, beyond care related to the reason for admission at the admission time.(8,9,18,21,22,24,19) In addition, de Castro *et al.*(24), Naidon *et al.,*(25) Pai, *et al.*(22) and Pell *et al.,*(18) include care for psychological needs. On the other hand,(7,16,25,26) add care for spiritual needs and others(19,21,26,27,16,23) emphasize care for social needs and Kavosi *et al.*(25) mention that communication by professionals is a significant part of comprehensive care, since it facilitates the relationship with the patient and his or her family, the understanding of his or her illness and the processes of examination, diagnosis and care planning; it weighs and moderates the expectations of healing, and allows the patient to be proactive and participate fully in all of them. In short, adequate communication humanises care and makes the patient the owner of his/her decision-making process.(16,19) On the other hand,(8,9,27,24) agree that the care plan is the best tool to provide comprehensive care and that nursing assessment allows for a patient examination in all its aspects, but they also highlight the need to continue the methodology and implementation training to increase our capacity and promptness when using them. 

*Assessment of Methodological Quality.* The selected articles were evaluated using the peer review technique independently. Each study was evaluated for minimum quality. The methodological quality assessment was found to be between moderate and optimal, with the exception of 4 papers with low quality*.*(^28^) *.*(^31^) 

## Discussion

This research study aimed to identify how the concept of "comprehensive care" in nursing care has been described from a professional point of view. After a systematic search, 14 documents were identified using an appropriate methodology; these documents were both qualitative and quantitative. The concept "Comprehensive Care" is used to refer to or describe various health services. Regarding the nursing profession, it is mainly used to refer to comprehensive nursing care techniques, protocols, programmes and plans, which cover care in all areas of the individual as a complement to or independently of the clinical needs arising from care.

In the results of the systematic search, the Nursing Care Process is highlighted as an implementing tool for care planning as reflected by Noia *et al*.(15) and Overcash *et al*.(32) This includes a vehicle that allows the provision of comprehensive care applied to the needs that diseases trigger in all areas of the individual. Nevertheless, the care plan can be considered as a hardly operative tool that needs some time of evaluation for the patient, in order to provide an effective care, as mentioned by Andre *et al*.,(8) Alvarenga *et al.,*(29) Lima Rocha *et al.,*(16) Peel *et al*.,(18) and Overcash *et al.,*(32) for whom patients were the object of study. 

Concerning the theoretical training, this should be focused on the health-disease process, diagnosis, treatment and approach to the patient in different circumstances, as sound scientific training improves and complements the nursing service, as ponted out by Overcash *et al.*(32) The technical specificity of the nursing practical skills is undeniable, as it is the importance of the theoretical biological support in the care for users. But the symbiosis between the other human conformations is also recognised, which at some point will coincide with the treatment, either to enhance it or to hinder it. Therefore, this outlook is presented from the beginning of training in academic life, highlighting for future professionals the integrating components of the paradigm and of the genuine human transcendence. Where the latter, even during physical illness, demonstrates elements that participate in the health continuum and spread beyond the homeostatic alteration.

On the other hand, it can be seen that nurses provide partial coverage of the care plan, since care in the psychosocial sphere is neglected due to the scarce inclusion of the family in the patient’s comprehensive care.(8) However, providing psychosocial and spiritual care is paramount, since this care impacts directly on the patient’s quality of life.(29) In addition, de Castro *et al*.(24) point out that good management of resources and services is necessary to achieve comprehensive care. It should be noted that, although at first glance they do not seem to be essential, nor do they seem to influence the patient’s clinical situation, well-being and quality of life are impaired without the necessary care in the long term, or there may be cases of somatisation and major clinical complications that delay the patient's healing and complete recovery. 

Providing comprehensive care for the individual in all aspects is a moral and ethical obligation for nursing, because it is based on the existence of meeting the needs that the disease triggers in people through the care plan, as an orderly nursing tool that allows diagnosis in all aspects and address the patient’s needs. Nencetti *et al.*(7) and Agyeman-Yeboah, *et al.*(20) agree on this. In addition, according to Agyeman-Yeboah *et al.,*(8) having a care plan allows the patient to recover faster, avoids complications or health problems, increases patient satisfaction and autonomy and, in economic terms, reduces health costs.

It should be noted that, as a whole, the different tools constitute a system that contemplates the whole being, providing care with changing and versatile components in their implementation that provide different guidelines for the nursing method of each patient. With regard to comprehensive care in the family and primary caregiver(19) (25) (28) emphasise that comprehensive care must include spiritual care and seek to focus on the patient's circumstances through prevention, promotion and health education in the individual, family and community, extending to all ages. 

This review supports reflective exercise where it is required to consider the patient and the primary caregiver within the interdisciplinary team, as they live and cope with the disease. This experience is useful and valuable during the different stages of the nursing method, giving a resolving nature demonstrated by modification of indicators. 

## Conclusion

Comprehensive care must be personalised and prioritise what the person needs most, even if it is not related to the clinical reason for admission. This care must be extended to the family and society. In addition, it should focus on spiritual care, social relations and personal projection, which are just as important as technical care, since their attention improves quality of life and patient satisfaction with the care received. Comprehensive care should be the lever that favours a balance between the patient's health, personal autonomy and satisfaction with his or her life in general terms.
